# Establishment of cell lines with porcine spermatogonial stem cell properties

**DOI:** 10.1186/s40104-020-00439-0

**Published:** 2020-04-10

**Authors:** Yi Zheng, Tongying Feng, Pengfei Zhang, Peipei Lei, Fuyuan Li, Wenxian Zeng

**Affiliations:** Key Laboratory for Animal Genetics, Breeding and Reproduction of Shaanxi Province, College of Animal Science and Technology, Northwest A&F University, Yangling, 712100 Shaanxi China

**Keywords:** Immortalization, Pig, Self-renewal, Spermatogonial stem cells, SV40 large T antigen

## Abstract

**Background:**

Spermatogonial stem cells (SSCs) are capable of both self-renewal and differentiation to mature functional spermatozoa, being the only adult stem cells in the males that can transmit genetic information to the next generation. Porcine SSCs hold great value in transgenic pig production and in establishment of porcine models for regenerative medicine. However, studies and applications of porcine SSCs have been greatly hampered by the low number of SSCs in the testis as well as the lack of an ideal stable long-term culture system to propagate porcine SSCs perpetually.

**Results:**

In the present study, by lentiviral transduction of plasmids expressing the simian virus 40 (SV40) large T antigen into porcine primary SSCs, we developed two immortalized cell lines with porcine SSC attributes. The established cell lines, with the expression of porcine SSC and germ cell markers UCHL1, PLZF, THY1, VASA and DAZL, could respond to retinoic acid (RA), and could colonize the recipient mouse testis without tumor formation after transplantation. The cell lines displayed infinite proliferation potential, and have now been cultured for more than 7 months and passaged for over 35 times without morphological abnormalities.

**Conclusions:**

We have for the first time established porcine SSC lines that could provide abundant cell sources for mechanistic studies on porcine SSC self-renewal and differentiation, thereby facilitating development of an optimal long-term culture system for porcine primary SSCs and their application to animal husbandry and medicine.

## Background

Spermatogenesis is an intricate process in the testes responsible for perpetual production of mature spermatozoa and therefore lifelong male fertility. Spermatogenesis is predicated on spermatogonial stem cells (SSCs). SSCs are capable of both self-renewal (to maintain the sufficient quantity) and differentiation (to mature functional spermatozoa), being the only adult stem cells in the males that can transmit genetic information to the next generation [1]. In clinics, SSC auto-transplantation in conjunction with germline genetic editing has the potential to treat male infertility resulted from spermatogenic failure and to prevent transmission of genetic diseases to the descendent [2, 3]. In animal husbandry, SSCs can be genetically modified by genome editing tools such as CRISPR-Cas9, and when combined with SSC allogenic transplantation, transgenic spermatozoa can be produced in the recipient testis, thereby providing a desirable means to generate animals with enhanced productivity and economic value [4].

Pigs (*Sus scrofa*) are a leading domestic species over the world for meat production. Also, they are increasingly exploited as an animal model in physiological and pharmacological research due to their high similarity to humans with respect to anatomy and physiology [5]. Porcine SSCs thus hold great value in transgenic pig production and in establishment of porcine models for regenerative medicine. Despite this, studies and applications of porcine SSCs have been greatly hampered by the low number of SSCs in the testis as well as little knowledge of the mechanisms underlying porcine SSC self-renewal and differentiation. To date, culture systems for murine SSCs have been well-established; murine SSCs can undergo exponential amplification *in vitro* for years without losing stem cell capacities [6–8]. However, long-term culture of SSCs from non-rodent species including pigs remains challenging. Recently, our group has for the first time established a culture system that could maintain the propagation of porcine SSCs for 2 months. After the 2-month culture period, cell proliferation came to a standstill, along with the prevalence of differentiation and apoptosis, leading to a sharp decline in the total cell number [9], suggesting that there is large room for improvement of porcine SSC culture.

Gaining more insights into the mechanisms underlying porcine SSC self-renewal and differentiation is a prerequisite for cell culture optimization. In this sense, establishment of an immortalized SSC line in pigs, as was accomplished in rodents [10, 11] years ago and in humans [12] recently, would provide ample cells for mechanistic studies, thereby facilitating development of an optimal culture system for porcine primary SSCs and, finally, their application to animal husbandry and medicine. To this end, here we employed lentiviral transduction to deliver plasmids expressing the simian virus 40 (SV40) large T antigen into early germ cells from 7-day-old pigs, and then enriched PLD6^+^ cells by FACS. Consistent with our recent report that PLD6 is a surface marker of porcine undifferentiated spermatogonia that can be used to enrich porcine SSCs with unprecedented efficiency [13], over 90% of the sorted PLD6^+^ cells were positive for porcine SSC markers lectin DBA [14], UCHL1, PLZF [15, 16], and the pan-germ cell marker VASA [17]. Later, we successfully established two immortalized cell lines that both expressed porcine SSC markers UCHL1, PLZF [15, 16] and THY1 [18], germ cell markers VASA and DAZL [17], as well as CXCR4, an essential factor involved in SSC survival and in homing to stem cell niche [19, 20]. The established cell lines with porcine SSC properties could offer abundant cell sources for the future fundamental studies.

## Methods

### Animals

Testis tissue was obtained from 7-day-old Landrance pigs in the Besun farm (Yangling, Shaanxi, China). The testis samples were maintained in Dulbecco’s phosphate-buffered saline (DPBS) upon castration and transported to the laboratory shortly. All animal procedures were in line with and approved by the animal ethical committee of Northwest A&F University.

### Isolation and preliminary enrichment of porcine SSCs

Isolation and preliminary enrichment of porcine SSCs were performed as previously reported [9, 21]. In brief, after removing the tunica albuginea and collective tissue, testes were minced into small pieces and incubated with collagenase type IV (2 mg/mL; Thermo Fisher Scientific) in Dulbecco’s modified eagle medium (DMEM, high glucose; Thermo Fisher Scientific) for 30 min at 37 °C. After centrifugation to remove Leydig cells and erythrocytes, the fragments of seminiferous tubules were incubated with 0.25% (w/v) trypsin-EDTA (Hyclone) and DNase I (0.1 mg/mL; Sigma-Aldrich) for 8 min at 37 °C. After filtering through a 40-μm mesh, the single-cell suspension was subjected to an optimized differential plating [9] to preliminarily enrich porcine SSCs. After overnight incubation, the floating and loosely attached cells, which contained a large proportion of SSCs, were collected for subsequent experiments.

### Immortalization and enrichment of porcine SSCs

Two lentiviral vectors, namely pLOX-Ttag-iresTK (addgene No. 12246) and Ef1a-Large T-Ires-Puro (addgene No. 18922), were purchased from addgene. Lentiviruses were produced by co-transfecting the either transfer vector and the 2^nd^ generation packaging vectors psPAX2 and pMD2.G into HEK293T cells, and were concentrated using ultracentrifugation. The porcine cells collected after differential plating were seeded to 12-well plates coated with laminin (20 μg/mL; Sigma-Aldrich), and transduced with the prepared lentiviruses by using “spinfection”. This method can be used to transduce SSCs with substantial efficiency [22]. Briefly, the cells exposed to 10 μg/mL polybrene (Sigma-Aldrich) and the concentrated virus supernatant at a multiplicity of infection (MOI) of 100 were centrifuged at 3,000×*g* for 1 h at 32 °C, followed by 16 h of incubation at 37 °C. One day after lentiviral transduction, cells were subjected to FACS employing an antibody against PLD6 (rabbit anti-PLD6; ab237612, Abcam). The sorted PLD6^+^ cells were seeded to 24-well plates coated with laminin (20 μg/mL; Sigma-Aldrich) for expansion.

### Porcine SSC culture

The sorted immortalized porcine SSCs were cultured in the complete medium made up of DMEM (high glucose; Thermo Fisher Scientific), 5% (v/v) fetal bovine serum (FBS; Thermo Fisher Scientific), 5% (v/v) knockout serum replacement (KSR; Thermo Fisher Scientific), 2 mmol/L Glutamax (Thermo Fisher Scientific), 1× non-essential amino acid (Thermo Fisher Scientific), 1 × vitamin solution (Thermo Fisher Scientific), 5 × 10^− 5^ mol/L 2-mercaptoethanol (Sigma-Aldrich), 1× penicillin-streptomycin (Hyclone), 20 ng/mL recombinant human GDNF (Peprotech), 40 ng/mL soluble GFRA1 (Peprotech) and 10 ng/mL recombinant human bFGF (Peprotech). The cells were refreshed every 2 d, and when reaching 80% of confluency, they were passaged to new wells coated with laminin (20 μg/mL; Sigma-Aldrich) at a ratio of 1: 2. The cells were maintained at 35 °C in an atmosphere of 5% CO_2_ in air. For retinoic acid (RA)-induced differentiation, cells were exposed to the growth factor-free medium harboring 5 μmol/L all-trans-RA (Sigma-Aldrich) for 2 d. In control groups, 0.1% ethanol was added to the medium as a vehicle.

### Immunocytochemistry

Immunocytochemical analysis was conducted on 4% paraformaldehyde (PFA)-fixed cytospin slides of sorted or cultured cells. For ɑ-tubulin and phalloidin staining, cells were cultured in 48-well plates and fixed with 4% PFA. The cells were permeabilized with 0.1% triton-X (Sigma-Aldrich) for 15 min at room temperature. After washing, the cells were blocked with 3% bovine serum albumin (BSA) for 1 h at room temperature, and later subjected to the primary antibody incubation at 4 °C overnight. The primary antibodies used were lectin DBA (1: 100; FL-1031, Vector Laboratories), mouse anti-UCHL1 (1: 200; ab8189, Abcam), rabbit anti-PLZF (1: 200; PA5–29213, Thermo Fisher Scientific), rabbit anti-VASA (1: 200; ab13840, Abcam), rabbit anti-DAZL (1: 100; ab34139, Abcam), goat anti-CXCR4 (1: 100; ab1670, Abcam), rabbit anti-SV40 (1: 200; 15,729, Cell Signaling Technology), mouse anti-PCNA (1: 200; sc-56, Santa Cruz Biotechnology), rabbit anti-ɑ-tubulin (1: 200; 11224–1-AP, Proteintech) and TRITC Phalloidin (1: 200; 40734ES75, Yeasen). For negative controls, the corresponding isotype IgGs were used in place of the primary antibodies. After washing on the next day, the cells were incubated with the corresponding donkey anti-rabbit/mouse/goat secondary antibodies (Alexa Fluor 488/594, 1: 500; Thermo Fisher Scientific) for 1 h at room temperature, followed by the nuclear staining with DAPI (1: 1,000; Bioworld Technology) for 5 min at room temperature. Digital images were captured with the Nikon Eclipse 80i fluorescence microscope camera. Percentages of sorted cells positive for porcine SSC and germ cell markers were calculated by the numbers of positive cells divided by the counted whole cell numbers. At least 300 cells were analyzed in each group.

### RT-PCR and q-PCR analyses

Total RNAs were extracted from testes and the cultured cells using Trizol (Thermo Fisher Scientific). After removing the genomic DNA by DNase (Qiagen) treatment, RNAs were reversely transcribed using the First Strand cDNA Synthesis Kit (Thermo Fisher Scientific), and PCR was then performed using the synthesized cDNAs as templates. Information of primers and the corresponding PCR products is depicted in Table 1. PCR products were separated by electrophoresis on 2% agarose gels, stained with ethidium bromide (EB) and visualized under the ultraviolet (UV) light. Total RNAs without reverse transcription (−RT) but with PCR were used as negative controls.

**Table 1:** Primer sequences for the RT-PCR and q-PCR analyses

Gene	Forward primer (5'→3')	Reverse primer (5'→3')	Product size, bp
*UCHL1*	CAGTAGCCAATAATCAGGAC	AAGGCATCCGACCATCAAG	249
*PLZF*	GCGGAAGACCTGGATGACCT	GTCGTCTGAGGCTTGGATGGT	105
*THY1*	TACCACCAACCTGCCCATTC	AGAAGTTGGTTCGAGAGCGG	116
*VASA*	TATGATGCGGGATGGAATAACTG	ACAGCTCTTACACAAGTCCCAA	144
*DAZL*	CCTTTGCTGACCCACAGTCT	GTGAGGGACTGGCCAATGAA	220
*CXCR4*	TCTACGCTTTCCTTGGAGCC	GAAGAATGTCCACCCCGCTT	115
*SV40*	AGAACAGCCCAGCCACTATAA	CCAAGCAACTCCAGCCAT	256
*PCNA*	GAACAGGAGTACAGCTGTGTA	CAGGCTCATTCATCTCTATGG	220
*GAPDH*	TCGGAGTGAACGGATTTGGC	TGACAAGCTTCCCGTTCTCC	189
*HPRT1*	TTATGCCGAGGATTTGGAAAAGGTT	ATCCAGCAGGTCAGCAAAGAAT	160
*KIT*	CATGCACCAATGAAGGCGGTT	CAGCCCGTGAGGGAGTAATT	166
*STRA8*	AGCCGTTTACTTTCACTCTGACC	GCTGTTTGCATTCCCATCCT	148

For q-PCR analysis, an IQ5 (Bio-Rad) platform was employed, and the reaction was conducted in a 25-μL volume system including the SYBR Green II PCR Mix (Takara). Reactions were run in triplicates from three independent experiments. *GAPDH* and *HPRT1* were used as reference genes, and the data were analyzed using the −ΔΔC_t_ method. Information of primers and the corresponding q-PCR products is depicted in Table 1.

### Western blot analysis

Protein samples were extracted from testes and the cultured cells using RIPA buffer and following the standard protocol. After quantification, proteins were separated by SDS-PAGE, and Western blot analysis was performed as previously described [23]. The primary antibodies used were mouse anti-UCHL1 (1: 1,000; ab8189, Abcam), rabbit anti-PLZF (1: 1,000; ab104854, Abcam), mouse anti-THY1 (1: 1,000; ab205719, Abcam), rabbit anti-VASA (1: 1,000; ab13840, Abcam), rabbit anti-DAZL (1: 500; ab34139, Abcam), goat anti-CXCR4 (1: 500; ab1670, Abcam), rabbit anti-SV40 (1: 1,000; 15,729, Cell Signaling Technology), mouse anti-PCNA (1: 1,000; sc-56, Santa Cruz Biotechnology), mouse anti-β-actin (1: 3,000; CW0096, CWBIO), rabbit anti-KIT (1: 1,000; 3074, Cell Signaling Technology) and rabbit anti-STRA8 (1: 1,000; ab49602, Abcam). The secondary antibodies used were goat anti-rabbit IgG-HRP (1: 2,000; CW0156, CWBIO), goat anti-mouse IgG-HRP (1: 3,000; CW0110, CWBIO) and rabbit anti-goat IgG-HRP (1: 3,000; CW0109, CWBIO). Finally, the blots were visualized with a Western Bright ECL Kit (Comwin) and chemiluminescence (Chemi-Doc XRS, Bio-Rad).

### Xenotransplantation

Overall 10 C57BL/6 J male mice at the age of 6-8 weeks (from the Fourth Military Medical University, Xi’an, China) received a single administration of 40 mg/kg body weight busulfan (Sigma-Aldrich) to deplete the endogenous germ cells. To exam the efficiency of busulfan treatment, one testis was removed in 1 month after the injection, followed by hematoxylin & eosin (H&E) staining on testis sections, as previously reported [23, 24]. To label the cells for transplantation, both cell lines at passage 20 were introduced with a vector expressing GFP (pGreenPuro; System Biosciences) by lentiviral transduction, following the aforementioned means. For each cell line, approximately 100,000 cells with the constitutive GFP expression were transplanted into one testis in 5 busulfan-treated mice via the efferent duct, following a standard protocol [25]. The other testis without transplantation in each individual served as the negative control. Two months after xenotransplantation, testes in the recipient mice were collected. After removing the tunica albuginea, the seminiferous tubules were dissected and dispersed with collagenase type IV (1 mg/mL; Thermo Fisher Scientific). Seminiferous tubules were then spread in 10 cm dishes with DPBS, and analyzed for colonization of transplanted GFP^+^ cells under a Nikon Eclipse 80i fluorescence microscope. The number of GFP^+^ colonies in each recipient testis was quantified. Subsequently, the dispersed seminiferous tubules were fixed in 4% PFA, and testis cryosections were made from two testes transplanted with Ttag and Puro-transduced cells, respectively. VASA and DAZL staining were performed on fixed seminiferous tubules and on testis cryosections, following standard procedures. SV40 staining was performed on fixed seminiferous tubules and its colocalization with GFP was visualized under a confocal laser scanning microscope (Andor).

### Karyotyping analysis

Both cell lines at passages 25-27 were used for karyotyping analysis. In brief, cells at 70% of confluency were treated with 100 ng/mL nocodazole for 16 h, swollen in 75 mmol/L KCl, fixed in methanol-glacial acetic acid (3: 1), and dropped onto chilled slides. After air-drying, slides were stained with DAPI and visualized under a Nikon Eclipse 80i fluorescence microscope. At least 100 cells were analyzed in each group.

### DNA content analysis

DNA content analysis by FACS was conducted as previously reported [26]. DNA content was analyzed with the FACS analyzer (BD Biosciences) and with the software ModFit LT (Verity Software House, USA).

### Statistics

All data were presented as the mean ± standard error of the mean (SEM) of three independent experiments unless otherwise stated. Statistical differences between groups were evaluated using the Student’s *t*-test (two-tail). A difference was considered statistically significant when *P* < 0.05 (*) and extremely significant when *P* < 0.01 (**).

## Results

### Immortalization and enrichment of porcine SSCs

**Fig. 1:**
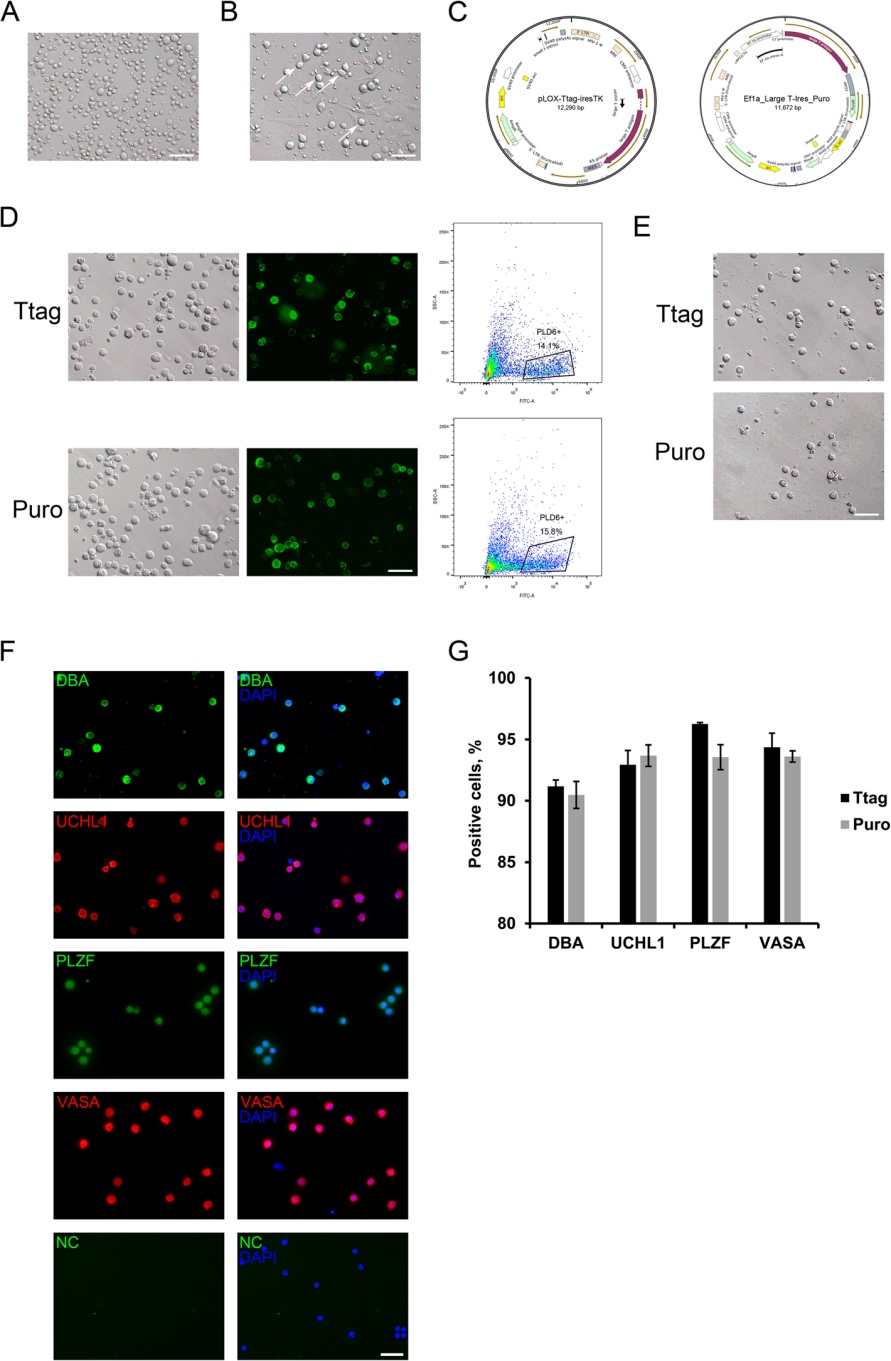
Immortalization and enrichment of porcine SSCs. **a** The single-cell suspension obtained after the two-enzymatic dissociation. Bar = 100 μm. **b** The cell fraction after differential plating. Arrows point to the putative germ cells. Bar = 100 μm. **c** Maps of the vector pLOX-Ttag-iresTK (hereafter referred to as Ttag) and Ef1a-Large T-Ires-Puro (hereafter referred to as Puro). **d** Left: living staining of Ttag- and Puro-transduced cells for PLD6. Bar = 100 μm; Right: enrichment of PLD6^+^ cells by FACS. **e** Images of Ttag- and Puro-transduced cells after sorting. Bar = 100 μm. **f** Staining of Ttag-transduced PLD6^+^ cells for lectin DBA, UCHL1, PLZF and VASA. NC: the negative control using the isotype IgG in place of the primary antibody. Bar = 50 μm. **g** Percentages of sorted cells positive for porcine SSC and germ cell markers. Data are presented as the mean ± SEM of three experiments (technical triplicates)

First, we collected testis tissue from 7-day-old piglets for germ cell isolation. After the two-enzymatic dissociation, we obtained the single cell-suspension comprising germ cells and somatic cells (Fig. 1a). By using our optimized differential plating, germ cells (Fig. 1b, arrows), characterized by their round shape and large diameter (around 10 μm), were clearly enriched in the cell population. The enriched germ cells were then separated to two fractions, and two distinct vectors expressing the SV40 large T antigen, namely pLOX-Ttag-iresTK (hereafter referred to as Ttag, Fig. 1c, left) and Ef1a-Large T-Ires-Puro (hereafter referred to as Puro, Fig. 1c, right), were introduced to two germ cell fractions respectively by the optimized lentiviral transduction (specified in Methods). The cells were infected with lentivirus for 16 h, and 24 h after lentiviral transduction, cells were subjected to FACS employing an antibody against PLD6. Before FACS, a portion of transduced cells were positive for PLD6 (Fig. 1d), and after FACS, the sorted PLD6^+^ cells exhibited the uniform morphology (Fig. 1e). Immunofluorescence analysis showed that over 90% of sorted cells were positive for porcine SSC markers lectin DBA, UCHL1, PLZF, and the pan-germ cell marker VASA (Fig. 1f, g and Fig. S1). Thus, we successfully enriched the immortalized porcine SSCs, and these cells were subjected to further culture.

### Immortalized porcine SSCs undergo gradual morphological transformation during long-term culture

**Fig. 2:**
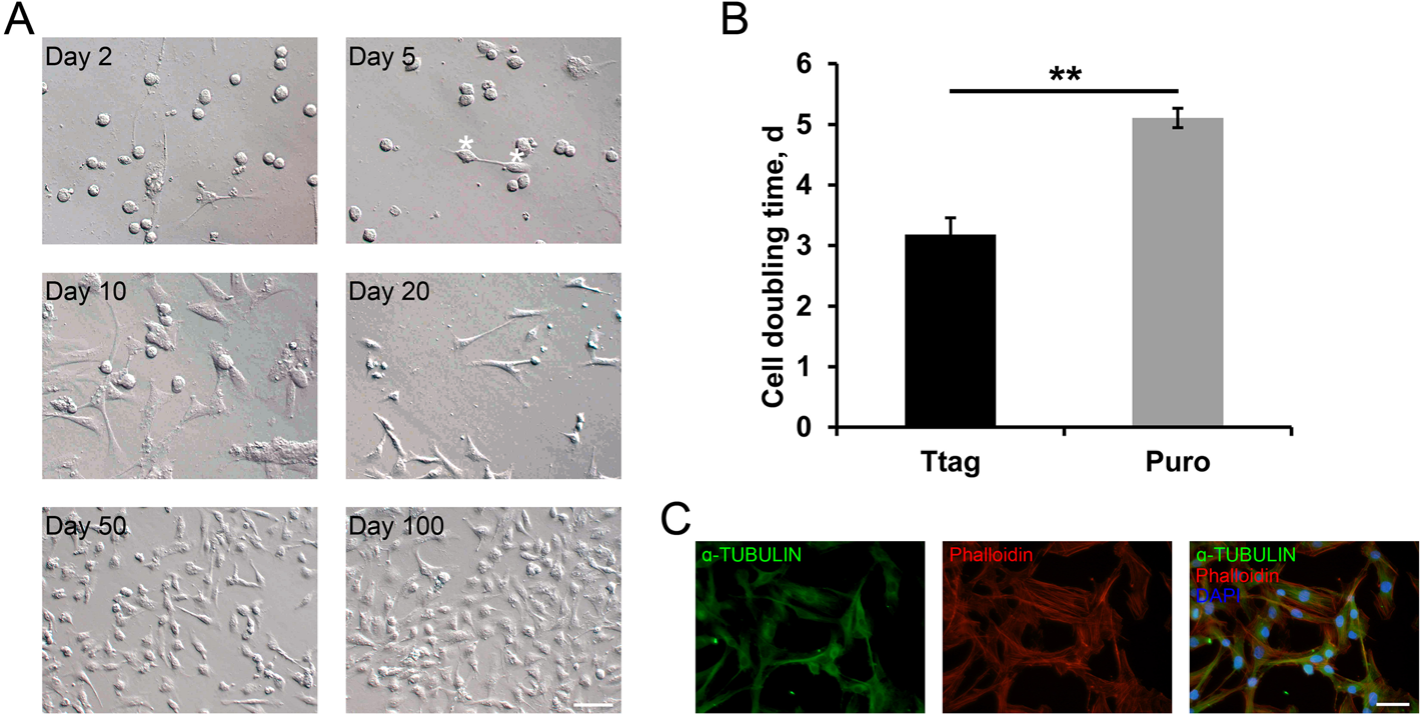
Immortalized porcine SSCs undergo gradual morphological transformation during long-term culture. **a** Representative images of the sorted Ttag-transduced SSCs on day 2, 5, 10, 20, 50 and day 100 after seeding. Asterisks indicate the cells that were experiencing morphological transformation. Bar = 100 μm. **b** Cell doubling time of SSCs transduced with Ttag or Puro. Data are presented as the mean ± SEM of three independent experiments. **: *P* < 0.01. **c** Staining of Ttag-transduced SSCs (passage 30) for ɑ-tubulin and phalloidin. Bar = 50 μm

We identified that the immortalized porcine SSCs gradually changed morphology during culture. Technically, on d 2 after seeding, the sorted Ttag-transduced porcine SSCs attached to laminin-coated 24-well plates, and remained a round/oval shape with a large nucleus. On d 5, a handful of cells started to transform, indicated by a somewhat elongated shape with cellular protrusion. On d 10, cells with a flattened and elongated shape became prevalent, even though some cells remained round/oval. On d 20, the round/oval cells were rarely observed. On d 50 and 100, the cells generally exhibited flattened morphology (Fig. 2a), very much similar to what has been described for SSC lines from mice, rats or humans. The sorted Puro-transduced porcine SSCs also underwent gradual morphological transformation and were indistinguishable from those transduced with Ttag (Fig. S2A). Despite that, the SSCs immortalized by Ttag showed the stronger proliferation, with a mean cell doubling time 3.18 d. By contrast, the doubling time of cells transduced with Puro was delayed up to 5.11 d (Fig. 2b). Nevertheless, both cell lines have now been cultured for more than 7 months, and passaged for over 35 times without any further morphological alterations or abnormalities. No polynucleated cells were observed, and the cytoskeleton appeared correct, as demonstrated by the immunofluorescence staining of ɑ-tubulin and phalloidin which binds to filamentous-actin (Fig. 2c and Fig. S2B).

### Characterization of immortalized porcine SSCs

**Fig. 3:**
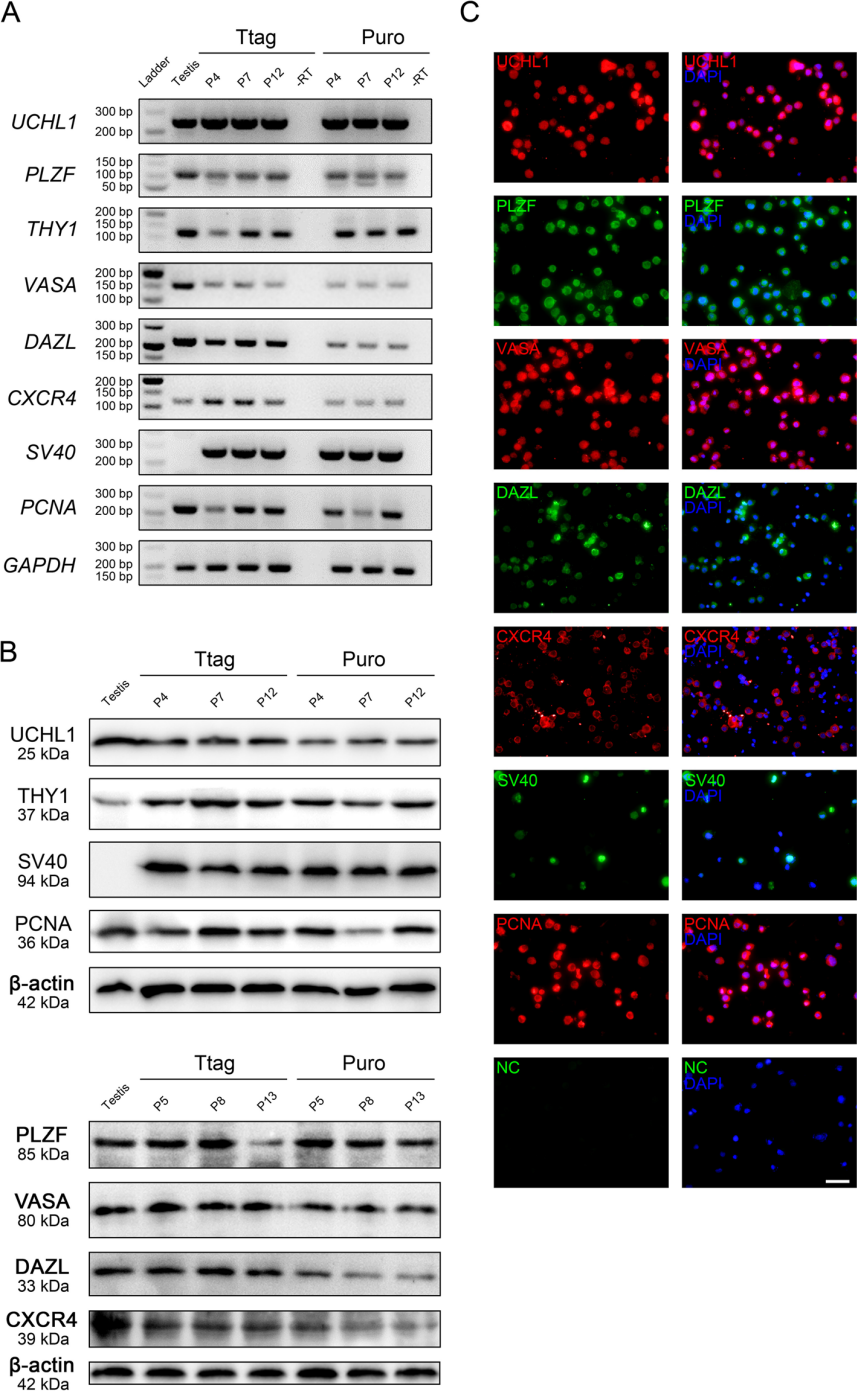
Characterization of immortalized porcine SSCs. **a** RT-PCR assay showing the transcripts of *UCHL1*, *PLZF*, *THY1*, *VASA*, *DAZL*, *CXCR4*, *SV40* and *PCNA* in the immortalized SSCs from passage 4 (P4) to passage 12 (P12). The RNAs from 7-day-old porcine testes are used as controls, and *GAPDH* serves as a loading control. -RT: the negative control omitting reverse transcription but with PCR reaction. **b** Western blot assay showing the protein expression of UCHL1, THY1, SV40, PCNA, PLZF, VASA, DAZL and CXCR4 in the immortalized SSCs at different passages. The protein extracts from 7-day-old porcine testes are used as controls, and β-actin serves as a loading control. **c** Staining of Ttag-transduced SSCs (passage 20) on cytospin slides for UCHL1, PLZF, VASA, DAZL, CXCR4, SV40 and PCNA. NC: the negative control using the isotype IgG in place of the primary antibody. Bar = 50 μm

To characterize and validate the immortalized porcine SSCs, we examined the expression of markers for porcine SSCs and germ cells throughout the culture, by using RT-PCR, Western blot and immunofluorescence. RT-PCR results showed that the transcripts of porcine SSC markers *UCHL1*, *PLZF* and *THY1* were present in the immortalized SSCs at different passages. As expected, pan-germ cell markers VASA and DAZL were expressed in both cell lines, thereby verifying their germ cell origin. In addition, CXCR4 was expressed in the cells. The mRNA expression of *SV40* and the proliferating cell nuclear antigen (*PCNA*) was also detected by RT-PCR (Fig. 3a), indicative of successful immortalization. Consistently, Western blot analysis demonstrated the expression of these markers at the protein level (Fig. 3b). Later, immunofluorescence assay performed on cytospin slides showed the expression of UCHL1, PLZF, VASA, DAZL, CXCR4, SV40 and PCNA in the immortalized SSCs at passage 20 (Fig. 3c and Fig. S3). Replacement of the primary antibodies by the corresponding isotype IgGs generated no signals. Overall, the results suggest that the established cell lines are porcine SSCs in phenotype.

### Immortalized porcine SSCs display limited differentiation in response to retinoic acid (RA)

**Fig. 4:**
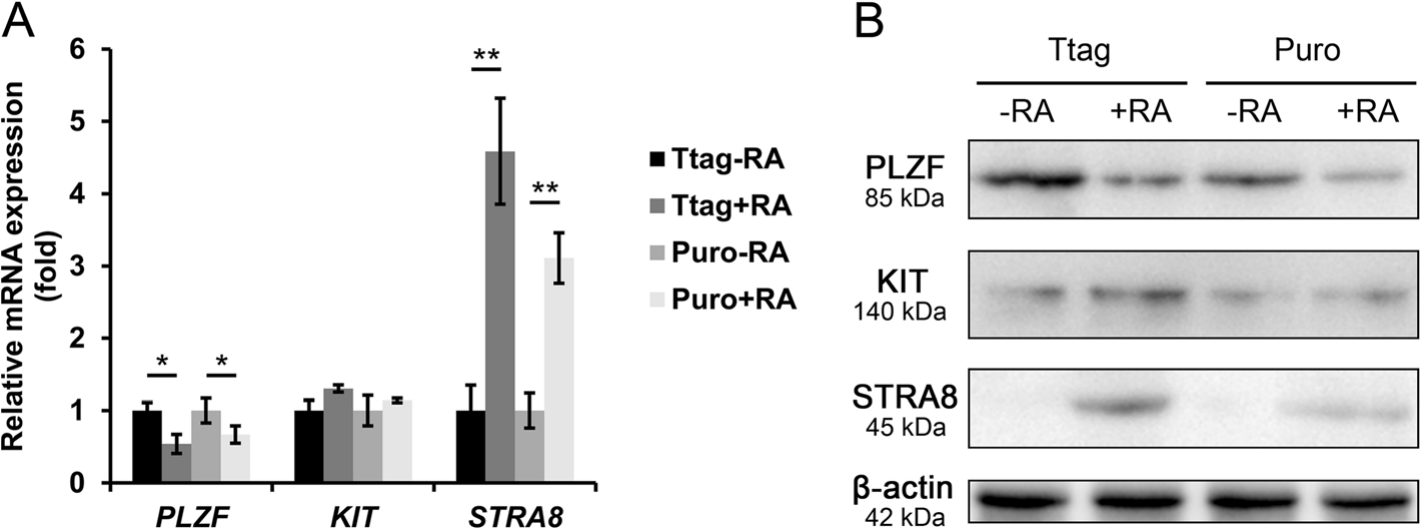
Immortalized porcine SSCs display limited differentiation in response to RA. **a** q-PCR analysis of *PLZF*, *KIT* and *STRA8* transcripts in immortalized porcine SSCs with/without RA treatment. Data are presented as the mean ± SEM of three independent experiments. *: *P* < 0.05; **: *P* < 0.01. **b** Western blot analysis of PLZF, KIT and STRA8 proteins in immortalized porcine SSCs with/without RA treatment. β-actin is used as a loading control

To investigate the differentiation potential of immortalized porcine SSCs, we treated both cell lines (passage 21) with RA for 2 d. q-PCR and Western blot analyses showed that RA treatment clearly upregulated STRA8, a gene activated by RA and crucial to spermatogonial differentiation and meiotic initiation. However, the expression of another differentiation marker KIT stabilized, and PLZF, a gene essential for SSC self-renewal, was only moderately downregulated by RA treatment (Fig. 4a, b). RA exposure had no influence on cell morphology. The results suggest that immortalized porcine SSCs could respond to RA but display limited differentiation.

### Immortalized porcine SSCs colonize the recipient mouse testis

**Fig. 5:**
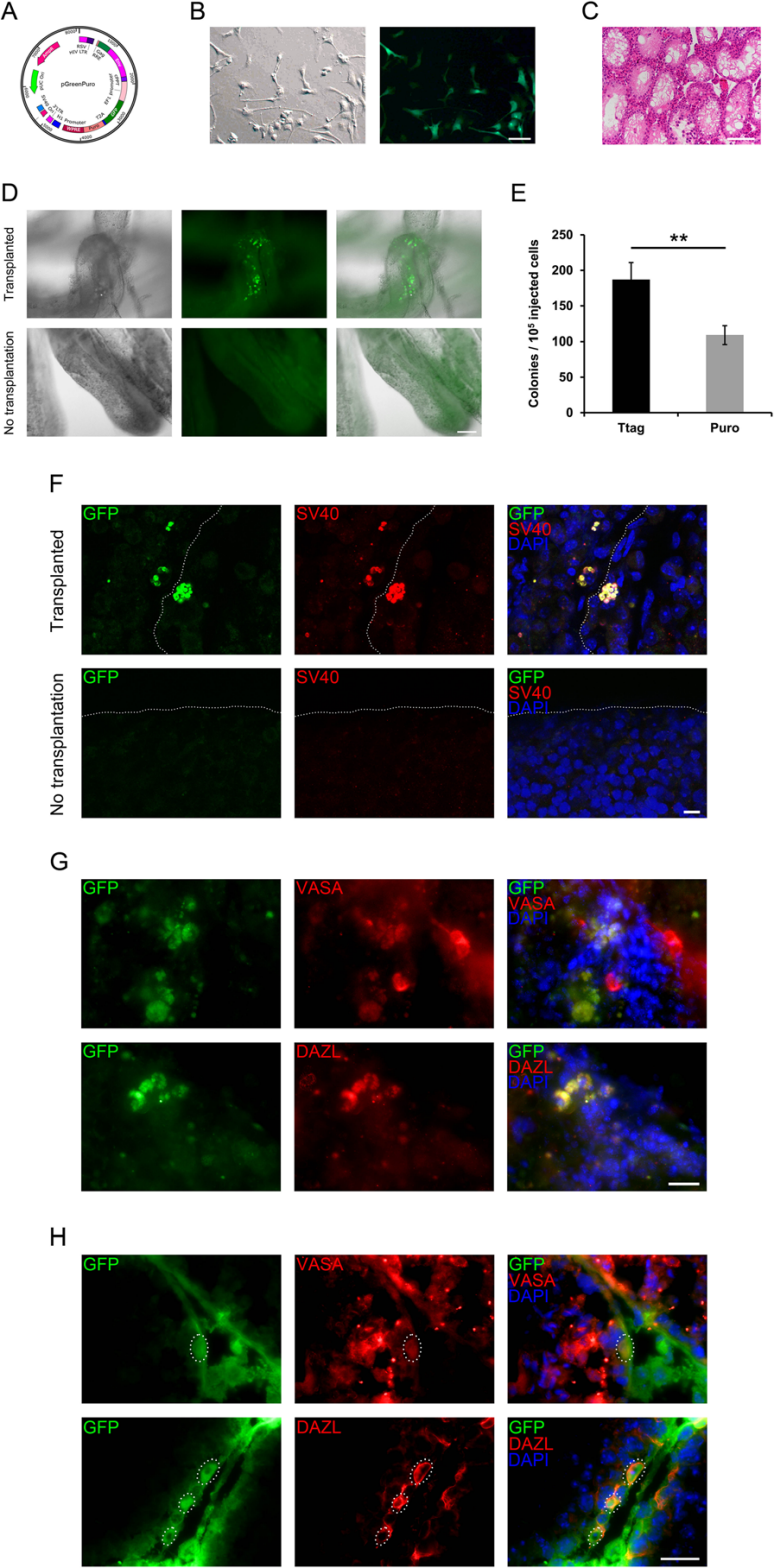
Ttag-transduced porcine SSCs colonize the recipient mouse testis. **a** The map of the vector pGreenPuro. **b** The bright (left) and fluorescent (right) images of Ttag-pGreenPuro-transduced cells (passage 20). Bar = 100 μm. **c** Hematoxylin & eosin (H&E) staining of testis sections from recipient mice 1 month after the busulfan treatment. Bar = 50 μm. **d** Visualization of the recipient seminiferous tubules (with/without transplantation of Ttag-pGreenPuro-transduced cells) under a bright (left) or fluorescent (middle) field. Bar = 50 μm. **e** The number of GFP^+^ cell colonies in the recipient testis. Data are presented as the mean ± SEM of four biological replicates (*n* = 4). **: *P* < 0.01. **f** The confocal microscopy analysis of the recipient seminiferous tubules (with or without transplantation of Ttag-pGreenPuro-transduced cells) showing the cell clusters co-expressing GFP and SV40. The dashed line delineates the putative basement membrane. Bar = 25 μm. **g**, **h** Staining of VASA and DAZL on dispersed seminiferous tubules **g** or on cryosections **h** from the testis transplanted with Ttag-transduced cells. Dashed ellipses refer to the transplanted cells settling down at the basement membrane. Bar = 20 μm

To demonstrate the stem cell attributes of the immortalized cell lines, we performed a transplantation assay that is currently viewed as a golden standard for bona fide SSCs. We first introduced a vector expressing GFP (Fig. 5a) into both cell lines (passage 20) by lentiviral transduction, to label the cells for transplantation. Then, for each cell line, approximately 100,000 cells with the constitutive GFP expression (Fig. 5b and Fig. S4A) were transplanted into one testis in 5 busulfan-treated mice (Fig. 5c). After 2 months, GFP^+^ cell colonies were discerned in seminiferous tubules of the transplanted testes, whist no GFP^+^ cells could be observed in the other recipient testes without transplantation (Fig. 5d and Fig. S4B). Quantification of the colony number per 100,000 injected cells indicated the more efficient colonization of Ttag-transduced cells than Puro-transduced cells (Fig. 5e). Later, we conducted the SV40 staining on dispersed recipient seminiferous tubules and visualized under a confocal laser scanning microscope. The confocal microscopy analysis revealed that cell clusters, which were composed of several round or oval cells co-expressing GFP and SV40, colonized the basement membrane of seminiferous tubules in the transplanted testis, while no GFP or SV40 expression could be observed in the other control testis without transplantation (Fig. 5f and Fig. S4C). To pinpoint the germ cell origin of the transplanted GFP^+^ cells, we additionally performed VASA and DAZL staining on dispersed seminiferous tubules (Fig. 5g and Fig. S4D) and on testis cryosections (Fig. 5h and Fig. S4E). The results clearly demonstrated that the transplanted GFP^+^ cells, which are of germ cell origin, could settle down at the basement membrane of seminiferous tubules. Notably, further developed cells positive for GFP could not be observed, and no tumor formation was found.

### Immortalized porcine SSCs display mutation in karyotype

**Fig. 6:**
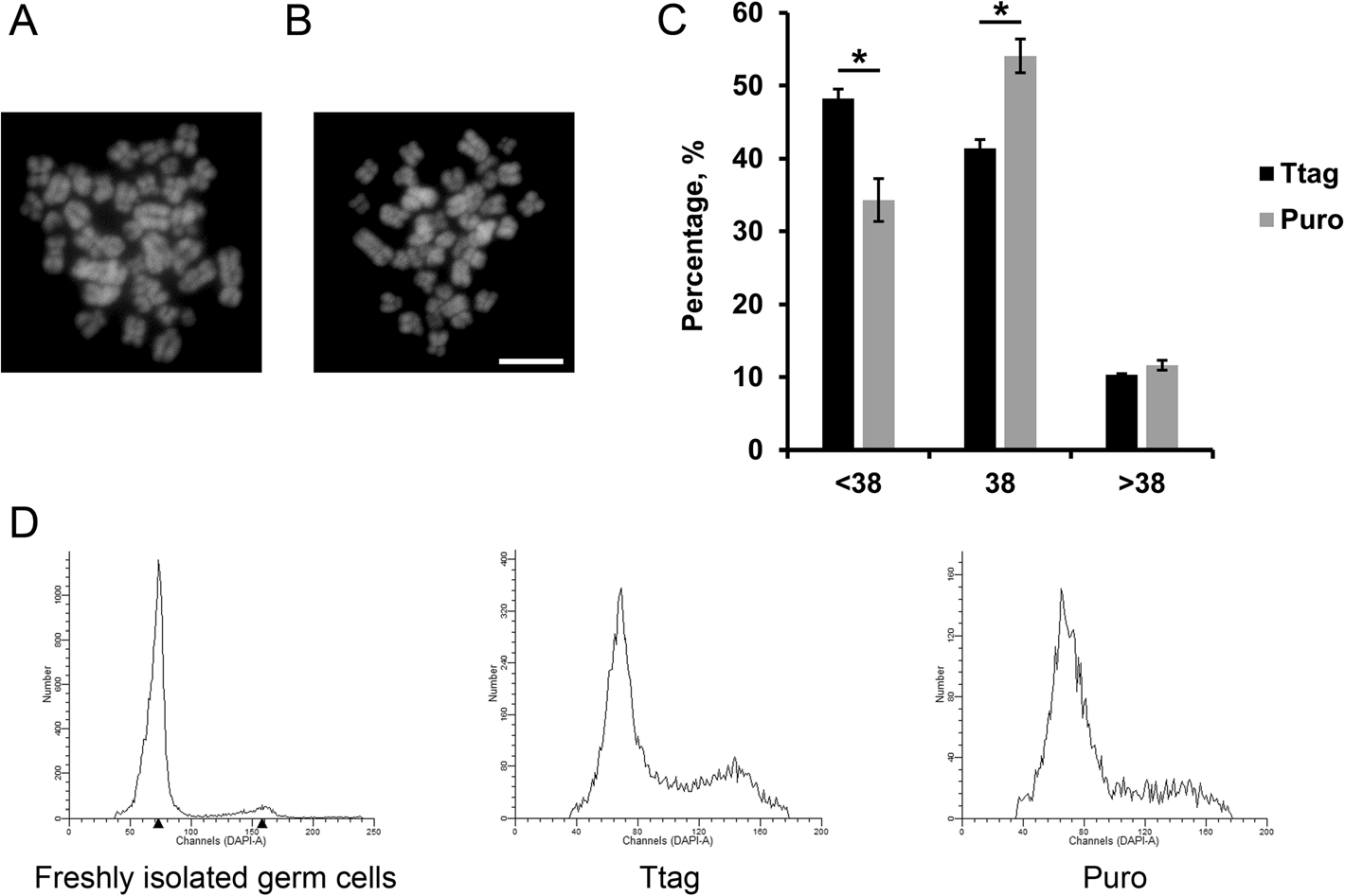
Immortalized porcine SSCs display mutation in karyotype. **a** An immortalized porcine SSC with a normal karyotype (*n* = 38). **b** An immortalized porcine SSC with an abnormal karyotype (*n* = 35). Bar = 5 μm. **c** Percentages of immortalized porcine SSCs with different karyotype, calculated by cell numbers with the defined karyotype divided by the counted whole cell numbers. Data are presented as the mean ± SEM of three independent experiments. *: *P* < 0.05. **d** DNA content analysis of freshly isolated porcine germ cells (after differential plating), Ttag-transduced SSCs (passage 33) and Puro-transduced SSCs (passage 31)

To examine whether immortalization by the SV40 large T antigen induces chromosomal aberrations in our established porcine SSC lines, we performed a karyotyping analysis for both cell lines at passages 25-27. We found that both cell lines displayed mutation in karyotype. Specifically, more cells immortalized by Puro remained a normal karyotype with 19 pairs of chromosomes, whereas a higher proportion of cells immortalized by Ttag showed chromosomal aberrations, in most cases chromosomal numerical reduction (Fig. 6a-c). Then, we employed FACS to measure the DNA content in both cell lines (Ttag at passage 33 and Puro at passage 31), with the freshly isolated porcine germ cells (after differential plating) as a control. Not surprisingly, for both cell lines substantially more cells were found in tetraploid peaks (Fig. 6d), indicating the altered DNA content.

## Discussion

Recently, we have for the first time developed a long-term culture system that could maintain the propagation of porcine primary SSCs for 2 months without losing stem cell characteristics [9]. However, this culture system is suboptimal and calls for improvement. Establishment of an immortalized porcine SSC line would provide sufficient cells for mechanistic studies on SSC self-renewal and differentiation, thereby contributing to optimization of the porcine primary SSC culture. Up to date, immortalized SSC lines from rodents [10, 11] and humans [12] have been developed by several groups, all taking advantage of the SV40 large T antigen. Therefore, we presumed that porcine SSCs could also be immortalized in this way. We first used our optimized differential plating [9] to preliminarily enrich porcine SSCs. Given that SSCs have been reported to be refractory to common transfection methods [27, 28], we next employed an optimized lentiviral transduction approach (spinfection) [22] to deliver the plasmids containing the SV40 large T antigen to SSCs. Then, to further enrich the transduced SSCs, we exploited FACS to sort PLD6^+^ cells. Our recent article has reported that PLD6 is a surface marker of porcine undifferentiated spermatogonia and that it can be used to enrich porcine SSCs with unprecedented efficiency [13]. In line with that study, the sorted PLD6^+^ cells displayed the uniform morphology and were almost all positive for porcine SSC markers lectin DBA, UCHL1, PLZF as well as the germ cell marker VASA, indicative of a highly pure immortalized cell population.

Previous studies have well demonstrated that primary SSCs from mammalian species tend to form typical grape/morula-like cell colonies *in vitro* [8, 9, 22, 29–31], while the immortalized mouse [11], rat [10] and human SSC lines [12] all display a flattened and elongated shape. Consistently, we identified that the immortalized porcine SSCs underwent gradual morphological transformation, switching from the round/oval shape to the flattened morphology after days of culture. The SV40 large T antigen may play a pivotal role in this process. Traditionally, the SV40 large T antigen is viewed as a robust agent for immortalization of animal cells [32]. The SV40 large T antigen can perturb the retinoblastoma susceptibility protein pRb and the tumor suppressor protein p53, two cell-cycle regulators that render cells enter a replicative senescent status. Moreover, the SV40 large T antigen is able to activate the telomerase activity in the transduced cells [33]. Nonetheless, the constitutive expression of the SV40 large T antigen, typically fulfilled by lentivirus-mediated integration into the host genome, has been reported to be a leading cause of cell transformation [34]. It is noticeable that the SV40 large T antigen-mediated cell transformation is often accompanied by chromosomal structural or numerical alterations [12, 35], which is exactly the case in our present study, as revealed by the karyotyping analysis. Transcriptional activators, e.g. p300 and CBP, may be involved in the process of cell immortalization and transformation [34].

Despite the morphological transformation, the immortalized cells expressed the porcine SSC markers UCHL1, PLZF and THY1 throughout the culture period, at both RNA and protein levels. Germ cell markers VASA and DAZL were also expressed, corroborating their germ cell origin. It is somewhat surprising that the cell line immortalized by Ttag showed the stronger proliferation than that immortalized by Puro, which might be due to the distinct promotors in the two vectors (CMV in Ttag and EF1ɑ in Puro) driving the SV40 large T antigen expression. CMV, while typically used to drive the ectopic expression of cassettes in a wide array of mammalian cells, is prone to be silenced in stem cells. Nagano et al. [36] reported that EF1ɑ but not CMV was efficient in attaining the ectopic expression in SSCs. Nevertheless, a recent study demonstrated the effective expression of the CRISPR-Cas9 cassettes driven by CMV in cultured mouse primary SSCs [28]. Thus, the influence of these two promotors on SSCs warrants systematic exploration in future. In spite of the differential growth rate, both cell lines have now been cultured for more than 7 months, and passaged for over 35 times without morphological abnormalities, becoming abundant cell sources for mechanistic studies on porcine SSC self-renewal and differentiation.

To investigate the differentiation potential of immortalized porcine SSCs, we treated both cell lines with RA. Previous reports have well demonstrated that RA can induce spermatogonial differentiation both *in vivo* and *in vitro* [31, 37–39]. Specifically, exposure of long-term cultured mouse SSCs to RA almost eradicated the expression of *Plzf* and *Oct4*, with significant upregulation of differentiation markers such as *Stra8* and *c-Kit*. Cell morphology also gradually changed during RA-induced *in vitro* differentiation [31, 37, 38]. Here, we found that immortalized porcine SSCs could respond to RA but displayed limited differentiation, characterized by less downregulation of *PLZF* and upregulation of *STRA8*, the stable *KIT* expression, as well as no apparent alteration in cell shape when compared to the primary mouse SSCs. The discrepancy might be ascribed to the constitutive expression of the SV40 large T antigen. Indeed, although it can immortalize cells and enable cells to propagate permanently, the SV40 large T antigen has been reported to trigger cell transformation and compromise cell differentiation [40].

To confirm the SSC properties of the established cell lines, they were transplanted into the testes of busulfan-treated mice. At present, the transplantation technique serves as a golden assay for functionality of SSCs. After injection into the recipient testis through efferent duct or rete testis, only SSCs are capable of going through the blood-testis barrier and migrating to the basement membrane of seminiferous tubules, from where they reinitiate self-renewal and differentiation, thereby reestablishing donor-derived spermatogenesis [1]. Here, we found that both cell lines were able to colonize the recipient mouse testis after xenotransplantation, demonstrating their SSC behavior. Quantification of the colony number indicated the more efficient colonization of Ttag-transduced cells. This, along with the stronger *in vitro* proliferation, suggest that SSC properties are better preserved in Ttag-transduced cells, and that this cell line might be preferable to the future mechanistic studies. Nonetheless, no further differentiation was observed for both cell lines in the recipient testes, similar to the transplanted rat [10] and human SSC line [12]. This is not unexpected for two reasons. First, while entire rat spermatogenesis could be fulfilled after xenotransplantation of rat SSCs into mouse testes [41, 42], it has been reported that the transplanted porcine SSCs did not undergo further development in mice. Presumably, the mouse SSC niche cannot support the differentiation of porcine SSCs, likely owing to the large phylogenetic distance between mice and pigs [43]. Second, the SV40 large T antigen is likely to deprive cells of the ability to differentiate [40]. An illustration of this point is that whilst the primary rat SSCs generated full spermatogenesis after xenotransplantation [41, 42], the immortalized rat SSC lines failed to undergo further differentiation in the recipient mouse testis [10]. Despite this, the colonization ability after transplantation remains a proof for the SSC property of our established cell lines.

## Conclusions

We have for the first time, to our knowledge, developed cell lines that not only express porcine SSC and germ cell markers but also can respond to RA and colonize the recipient mouse testis without tumor formation after transplantation. Thus, the established cell lines hold porcine SSC attributes, and will provide sufficient cell sources for mechanistic studies on porcine SSC self-renewal and differentiation, thereby facilitating development of an optimal long-term culture system for porcine primary SSCs and, in the long run, their application to animal husbandry and medicine.

## Supplementary information

**Additional file 1: Figure S1.** Staining of Puro-transduced PLD6^+^ cells for lectin DBA, UCHL1, PLZF and VASA. NC: the negative control using the isotype IgG in place of the primary antibody. Bar = 50 μm.

**Additional file 2: Figure S2.** Puro-transduced porcine SSCs undergo gradual morphological transformation during long-term culture. **a** Representative images of the sorted Puro-transduced SSCs on day 2, day 5, day 10, day 20, day 50 and day 100 after seeding. Asterisks indicate the cells that were experiencing morphological transformation. Bar = 100 μm. **b** Staining of Puro-transduced SSCs (passage 30) for ɑ-tubulin and phalloidin. Bar = 50 μm.

**Additional file 3: Figure S3.** Staining of Puro-transduced SSCs (passage 20) on cytospin slides for UCHL1, PLZF, VASA, DAZL, CXCR4, SV40 and PCNA. NC: the negative control using the isotype IgG in place of the primary antibody. Bar = 50 μm.

**Additional file 4: Figure S4.** Puro-transduced porcine SSCs colonize the recipient mouse testis. **a** The bright (left) and fluorescent (right) images of Puro-pGreenPuro-transduced cells (passage 20). Bar = 100 μm. **b** Visualization of the recipient seminiferous tubules (with/without transplantation of Puro-pGreenPuro-transduced cells) under a bright (left) or fluorescent (middle) field. Bar = 50 μm. **c** The confocal microscopy analysis of the recipient seminiferous tubules (with or without transplantation of Puro-pGreenPuro-transduced cells) showing the cell clusters co-expressing GFP and SV40. The dashed line delineates the putative basement membrane. Bar = 25 μm. **d, e** Staining of VASA and DAZL on dispersed seminiferous tubules **d** or on cryosections **e** from the testis transplanted with Puro-transduced cells. Dashed ellipses refer to the transplanted cells settling down at the basement membrane. Bar = 20 μm.

## Data Availability

All data supporting our findings are included in the manuscript.
