# Supplementary figures and images for: Establishment of cell lines with porcine spermatogonial stem cell properties
Source: J Anim Sci Biotechnol. 2020 Apr 10;11:33. doi: 10.1186/s40104-020-00439-0 (PMC7146966; doi:10.1186/s40104-020-00439-0)

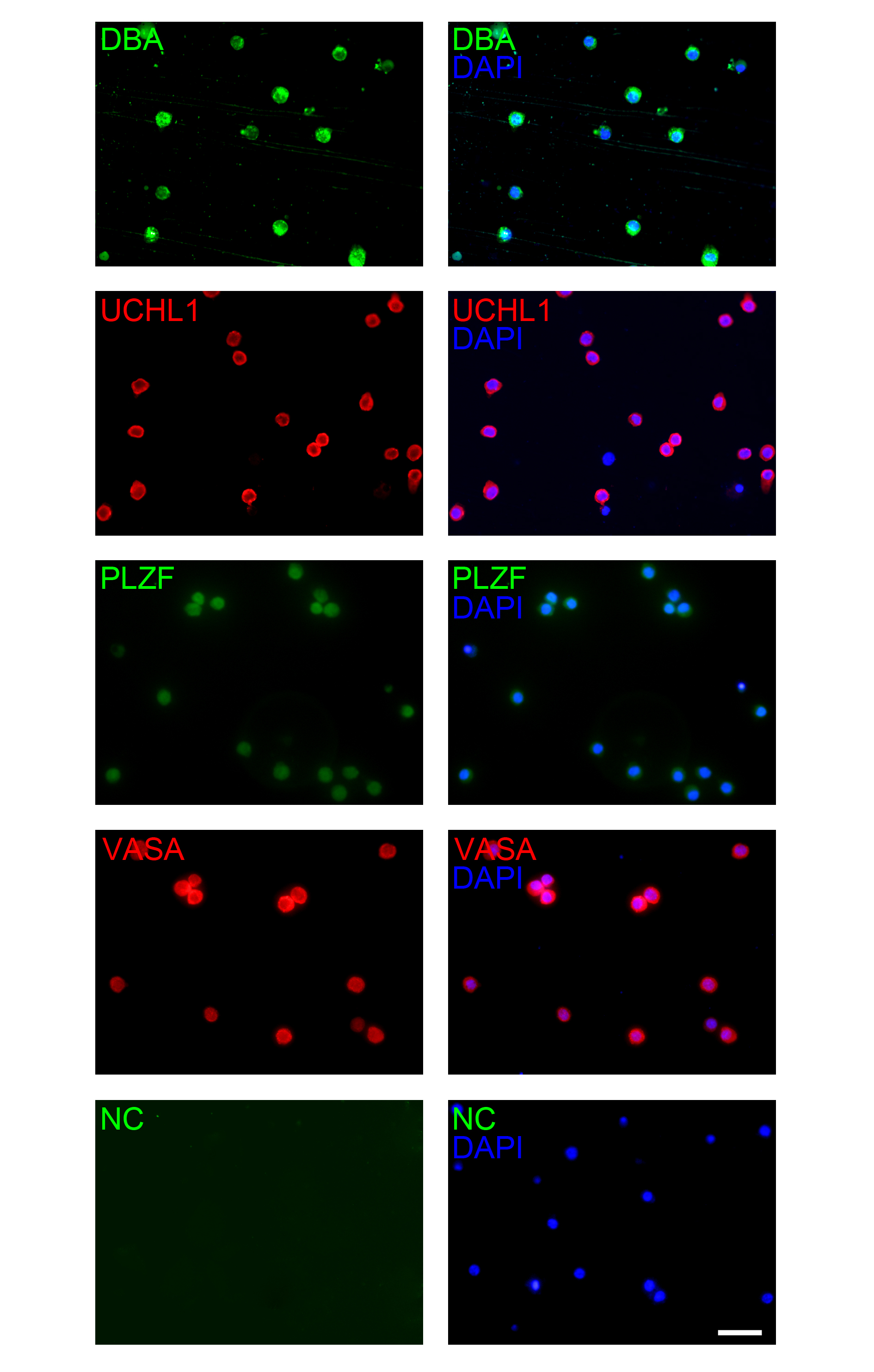

Supplement: Supplementary file 1 — Additional file 1: Figure S1. Staining of Puro-transduced PLD6+ cells for lectin DBA, UCHL1, PLZF and VASA. NC: the negative control using the isotype IgG in place of the primary antibody. Bar = 50 μm. [file 40104_2020_439_MOESM1_ESM.jpg]

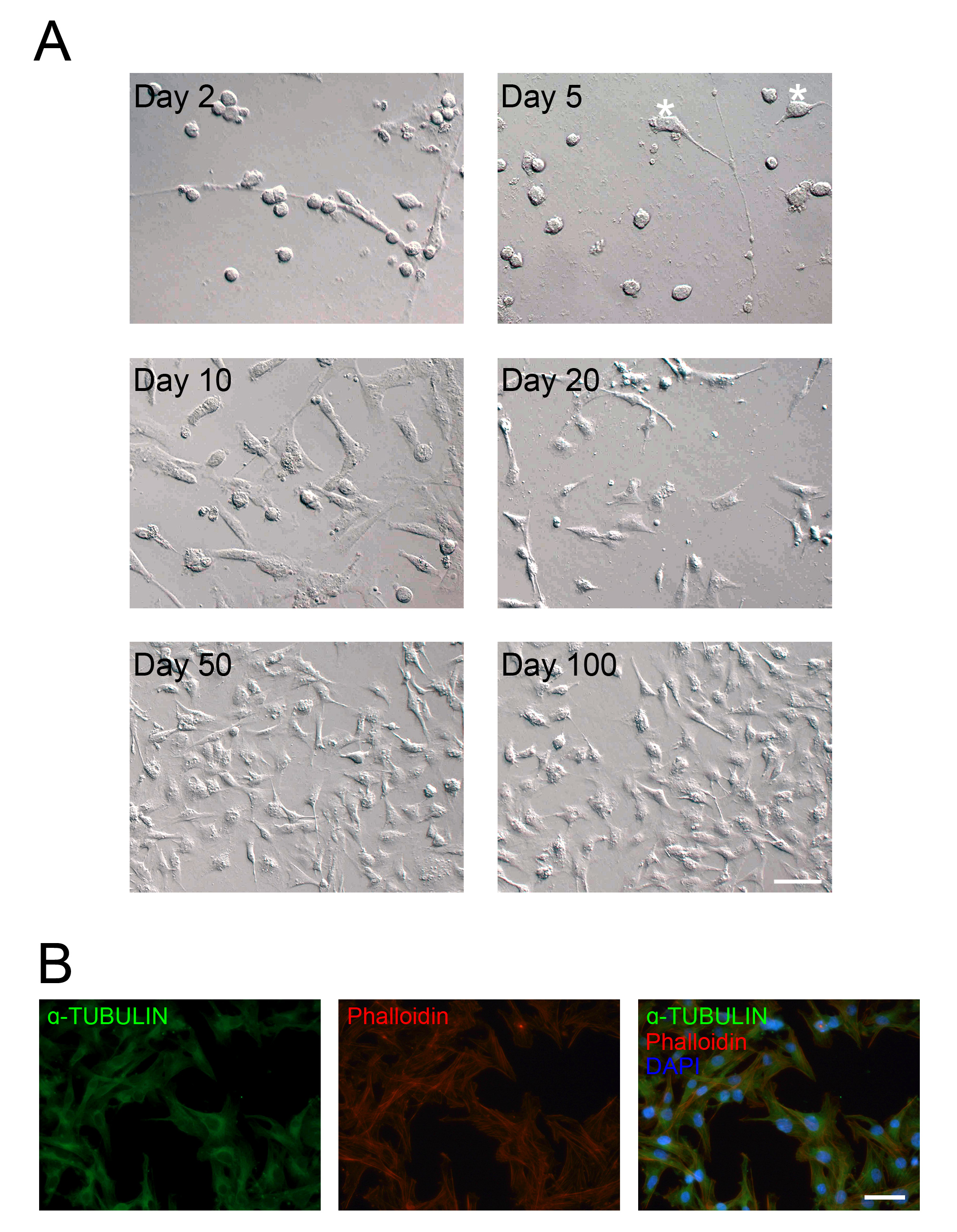

Supplement: Supplementary file 2 — Additional file 2: Figure S2. Puro-transduced porcine SSCs undergo gradual morphological transformation during long-term culture. a Representative images of the sorted Puro-transduced SSCs on day 2, day 5, day 10, day 20, day 50 and day 100 after seeding. Asterisks indicate the cells that were experiencing morphological transformation. Bar = 100 μm. b Staining of Puro-transduced SSCs (passage 30) for ɑ-tubulin and phalloidin. Bar = 50 μm. [file 40104_2020_439_MOESM2_ESM.jpg]

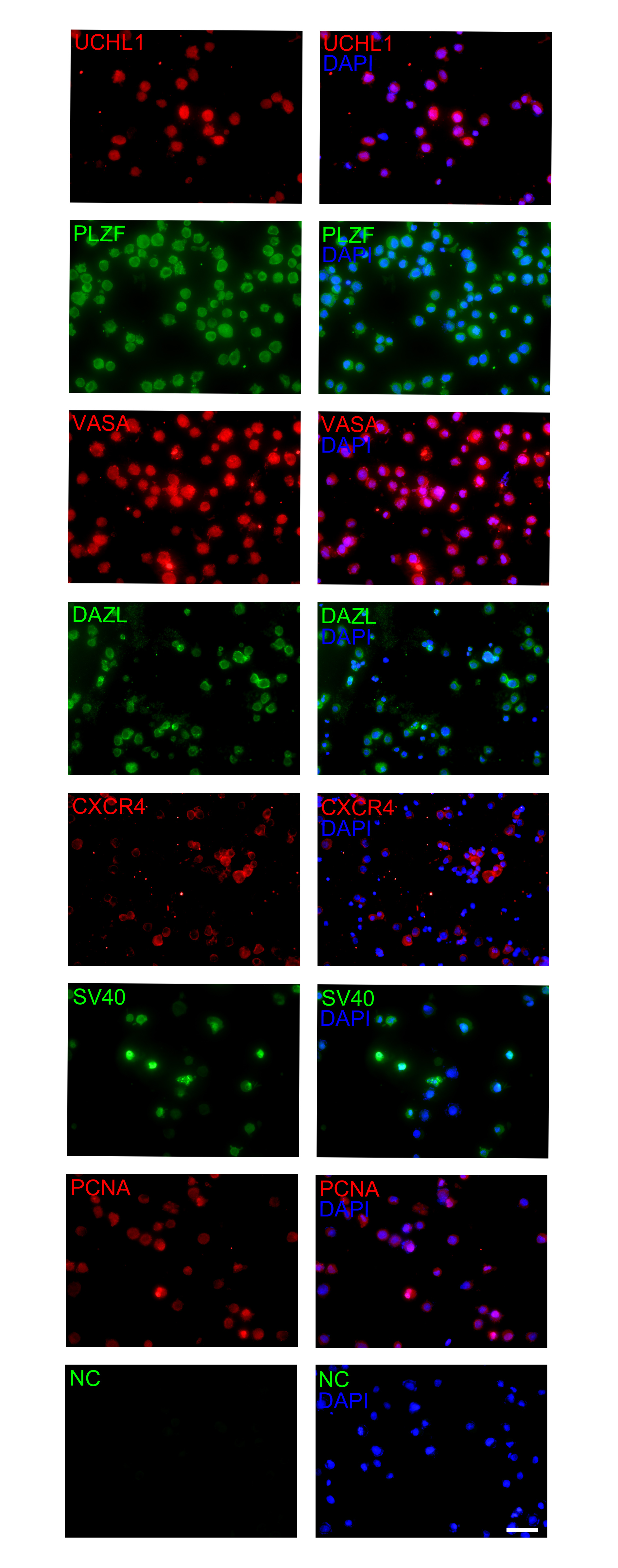

Supplement: Supplementary file 3 — Additional file 3: Figure S3. Staining of Puro-transduced SSCs (passage 20) on cytospin slides for UCHL1, PLZF, VASA, DAZL, CXCR4, SV40 and PCNA. NC: the negative control using the isotype IgG in place of the primary antibody. Bar = 50 μm. [file 40104_2020_439_MOESM3_ESM.jpg]

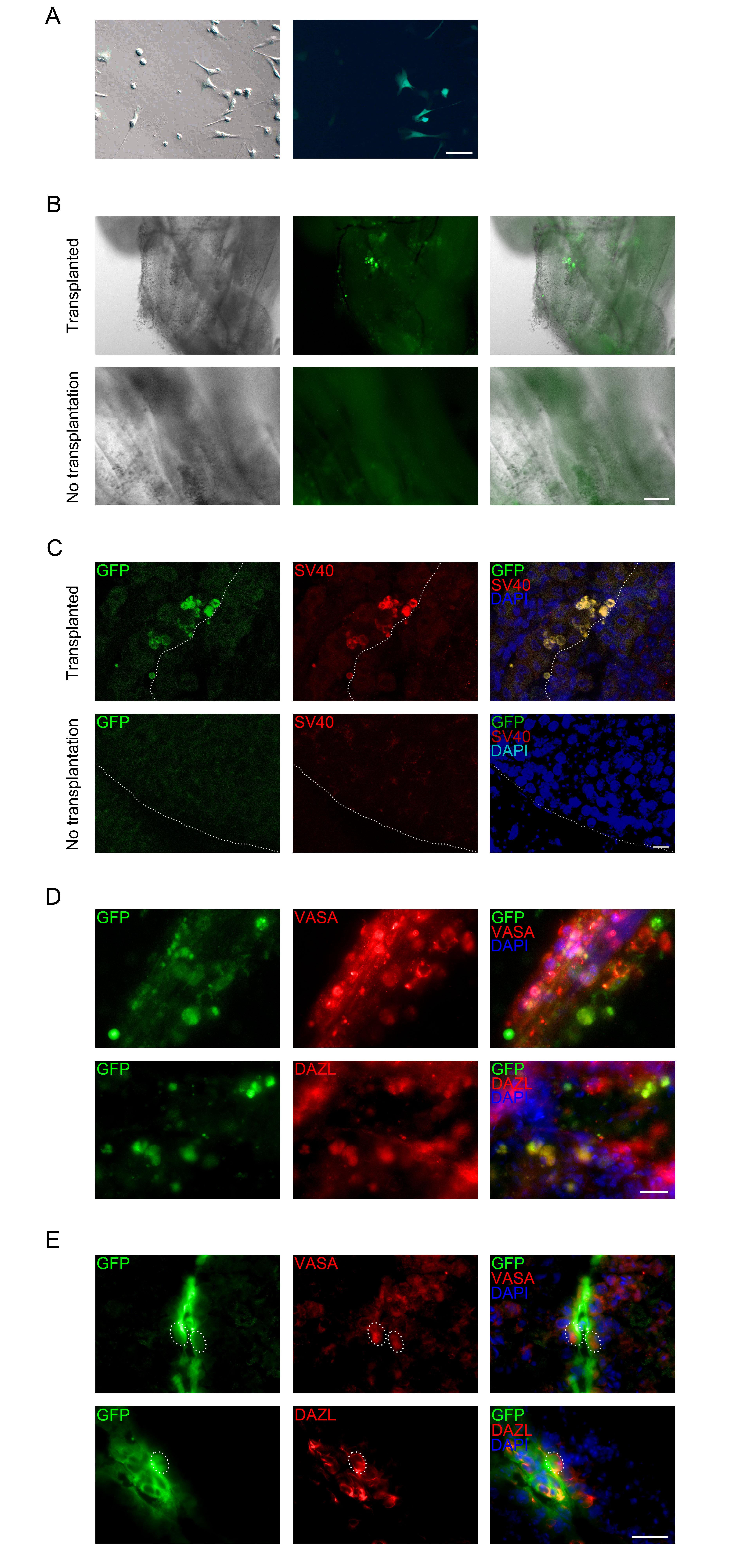

Supplement: Supplementary file 4 — Additional file 4: Figure S4. Puro-transduced porcine SSCs colonize the recipient mouse testis. a The bright (left) and fluorescent (right) images of Puro-pGreenPuro-transduced cells (passage 20). Bar = 100 μm. b Visualization of the recipient seminiferous tubules (with/without transplantation of Puro-pGreenPuro-transduced cells) under a bright (left) or fluorescent (middle) field. Bar = 50 μm. c The confocal microscopy analysis of the recipient seminiferous tubules (with or without transplantation of Puro-pGreenPuro-transduced cells) showing the cell clusters co-expressing GFP and SV40. The dashed line delineates the putative basement membrane. Bar = 25 μm. d, e Staining of VASA and DAZL on dispersed seminiferous tubules d or on cryosections e from the testis transplanted with Puro-transduced cells. Dashed ellipses refer to the transplanted cells settling down at the basement membrane. Bar = 20 μm. [file 40104_2020_439_MOESM4_ESM.jpg]
